# 3-Benzyl­sulfanyl-1*H*-1,2,4-triazol-5-amine

**DOI:** 10.1107/S1600536811052159

**Published:** 2011-12-10

**Authors:** Shuai Zhang, Pei-Jiang Liu, Dong-Sheng Ma, Guang-Feng Hou

**Affiliations:** a College of Chemistry and Materials Science, Heilongjiang University, Harbin 150080, People’s Republic of China

## Abstract

In the title mol­ecule, C_9_H_10_N_4_S, the dihedral angle between the benzene and triazole rings is 81.05 (5)°. In the crystal, N—H⋯N hydrogen bonds link the mol­ecules into infinite zigzag chains along [010].

## Related literature

For the biological properties of 1,2,4-triazoles derivatives, see: Paulvannan *et al.* (2001 ▶); El-Sagheer & Brown (2011 ▶).
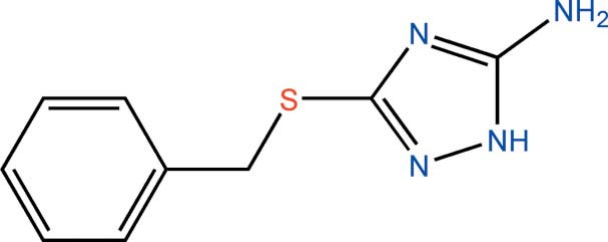


## Experimental

### 

#### Crystal data

C_9_H_10_N_4_S
*M* *_r_* = 206.27
Monoclinic, 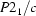

*a* = 9.870 (2) Å
*b* = 9.6370 (19) Å
*c* = 10.398 (2) Å
β = 90.18 (3)°
*V* = 989.0 (3) Å^3^
*Z* = 4
Mo *K*α radiation
μ = 0.29 mm^−1^
*T* = 293 K
0.38 × 0.26 × 0.11 mm

#### Data collection

Rigaku R-AXIS RAPID diffractometer
Absorption correction: multi-scan (*ABSCOR*; Higashi, 1995 ▶) *T*_min_ = 0.897, *T*_max_ = 0.970
9367 measured reflections
2267 independent reflections
1372 reflections with *I* > 2σ(*I*)
*R*_int_ = 0.059

#### Refinement

*R*[*F* ^2^ > 2σ(*F* ^2^)] = 0.047
*wR*(*F* ^2^) = 0.111
*S* = 1.04
2267 reflections
136 parameters
3 restraints
H atoms treated by a mixture of independent and constrained refinement
Δρ_max_ = 0.26 e Å^−3^
Δρ_min_ = −0.25 e Å^−3^

### 

Data collection: *RAPID-AUTO* (Rigaku, 1998 ▶); cell refinement: *RAPID-AUTO*; data reduction: *CrystalClear* (Rigaku/MSC, 2002 ▶); program(s) used to solve structure: *SHELXS97* (Sheldrick, 2008 ▶); program(s) used to refine structure: *SHELXL97* (Sheldrick, 2008 ▶); molecular graphics: *SHELXTL* (Sheldrick, 2008 ▶); software used to prepare material for publication: *SHELXL97*.

## Supplementary Material

Crystal structure: contains datablock(s) I, global. DOI: 10.1107/S1600536811052159/cv5211sup1.cif

Structure factors: contains datablock(s) I. DOI: 10.1107/S1600536811052159/cv5211Isup2.hkl

Supplementary material file. DOI: 10.1107/S1600536811052159/cv5211Isup3.cml

Additional supplementary materials:  crystallographic information; 3D view; checkCIF report

## Figures and Tables

**Table 1:** Hydrogen-bond geometry (Å, °)

*D*—H⋯*A*	*D*—H	H⋯*A*	*D*⋯*A*	*D*—H⋯*A*
N4—H41⋯N1^i^	0.89 (1)	2.20 (2)	3.044 (3)	158 (3)
N2—H21⋯N3^ii^	0.90 (1)	2.03 (2)	2.873 (2)	155 (2)

Symmetry codes: (i) 
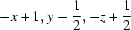
; (ii) 
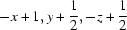
.

**Fig. 1.**
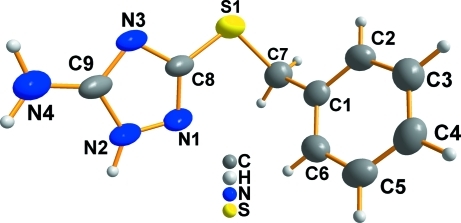
The molecular structure of (I) showing the atomic numbering and 30% probabilty displacement ellipsoids.

**Fig. 2.**
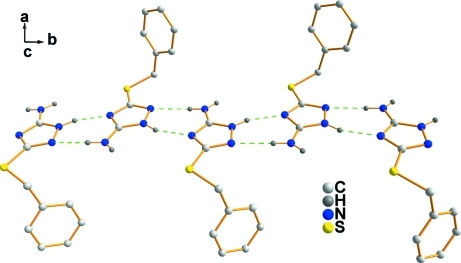
A portion of the crystal packing showing hydrogen-bonded (dashed lines) chains. C-bound H atoms omitted for clarity.

## supplementary crystallographic information

### Comment

We are paying attention to the synthesis and applications of substituted 1,2,4-triazoles due to their comprehensive biological activities such as antihypertensive, antifungal and antibacterial properties (Paulvannan *et al.*, 2001; El-Sagheer *et al.*, 2011). Herein, we report the synthesis and crystal structure of the title compound (I).

In (I) (Fig. 1), the benzene and triazole rings form a dihedral angle of 81.05 (5)°. In the crystal packing, the N—H···N hydrogen bonds (Table 1) link adjacent molecules into infinite zigzag chains along [010] (Fig. 2).

### Experimental

In aqueous (10 mL) solution of NaOH (0.40 g, 0.010 mol) and 5-amino-1*H*-1,2,4-triazole-3-thiol (1.16 g, 0.010 mol, commercial product) was added an ethanol (20 mL) solution of benzyl chloride (1.52 g, 0.012 mol) with stirring. After stirring for 10 min, the solution evaporated. Then the resulting precipitate was filtered off, diluted with water and purified to give the title compound. Colourless prismatic crystal suitable for X-ray analysis were obtained by recrystallization in chloroform solution.

### Refinement

H atoms bound to C atoms were placed in calculated positions and treated as riding on their parent atoms, with C—H = 0.93 Å (aromatic); C—H = 0.97 Å (methylene), and with *U*_iso_(H) = 1.2*U*_eq_(C). N-bound H atom were found from Fourier map, and were refined with restraint N—H = 0.90 (1) Å and *U*_iso_(H) = 1.5*U*_eq_(N).

### Crystal data

**Table:**

C_9_H_10_N_4_S	*F*(000) = 432
*M**_r_* = 206.27	*D*_x_ = 1.385 Mg m^−^^3^
Monoclinic, *P*2_1_/*c*	Mo *K*α radiation, λ = 0.71073 Å
Hall symbol: -P 2ybc	Cell parameters from 6282 reflections
*a* = 9.870 (2) Å	θ = 3.5–27.5°
*b* = 9.6370 (19) Å	µ = 0.29 mm^−^^1^
*c* = 10.398 (2) Å	*T* = 293 K
β = 90.18 (3)°	Block, colorless
*V* = 989.0 (3) Å^3^	0.38 × 0.26 × 0.11 mm
*Z* = 4	

### Data collection

**Table:**

Rigaku R-AXIS RAPID diffractometer	2267 independent reflections
Radiation source: fine-focus sealed tube	1372 reflections with *I* > 2σ(*I*)
graphite	*R*_int_ = 0.059
ω scan	θ_max_ = 27.5°, θ_min_ = 3.5°
Absorption correction: multi-scan (*ABSCOR*; Higashi, 1995)	*h* = −12→12
*T*_min_ = 0.897, *T*_max_ = 0.970	*k* = −12→12
9367 measured reflections	*l* = −13→13

### Refinement

**Table:**

Refinement on *F*^2^	Primary atom site location: structure-invariant direct methods
Least-squares matrix: full	Secondary atom site location: difference Fourier map
*R*[*F*^2^ > 2σ(*F*^2^)] = 0.047	Hydrogen site location: inferred from neighbouring sites
*wR*(*F*^2^) = 0.111	H atoms treated by a mixture of independent and constrained refinement
*S* = 1.04	*w* = 1/[σ^2^(*F*_o_^2^) + (0.0455*P*)^2^ + 0.1013*P*] where *P* = (*F*_o_^2^ + 2*F*_c_^2^)/3
2267 reflections	(Δ/σ)_max_ < 0.001
136 parameters	Δρ_max_ = 0.26 e Å^−^^3^
3 restraints	Δρ_min_ = −0.25 e Å^−^^3^

### Special details

**Table:**

Geometry. All esds (except the esd in the dihedral angle between two l.s. planes) are estimated using the full covariance matrix. The cell esds are taken into account individually in the estimation of esds in distances, angles and torsion angles; correlations between esds in cell parameters are only used when they are defined by crystal symmetry. An approximate (isotropic) treatment of cell esds is used for estimating esds involving l.s. planes.
Refinement. Refinement of F^2^ against ALL reflections. The weighted R-factor wR and goodness of fit S are based on F^2^, conventional R-factors R are based on F, with F set to zero for negative F^2^. The threshold expression of F^2^ > 2sigma(F^2^) is used only for calculating R-factors(gt) etc. and is not relevant to the choice of reflections for refinement. R-factors based on F^2^ are statistically about twice as large as those based on F, and R- factors based on ALL data will be even larger.

### Fractional atomic coordinates and isotropic or equivalent isotropic displacement parameters (Å^2^)

**Table:**

	*x*	*y*	*z*	*U*_iso_*/*U*_eq_	
C1	0.9298 (2)	0.2955 (2)	0.45864 (19)	0.0424 (5)	
C2	1.0473 (2)	0.2171 (2)	0.4593 (2)	0.0510 (6)	
H2	1.0536	0.1402	0.5131	0.061*	
C3	1.1549 (3)	0.2511 (3)	0.3816 (2)	0.0598 (7)	
H3	1.2329	0.1969	0.3823	0.072*	
C4	1.1468 (3)	0.3657 (3)	0.3029 (2)	0.0606 (7)	
H4	1.2198	0.3896	0.2510	0.073*	
C5	1.0312 (3)	0.4446 (3)	0.3008 (2)	0.0564 (6)	
H5	1.0256	0.5214	0.2469	0.068*	
C6	0.9232 (2)	0.4102 (2)	0.3784 (2)	0.0501 (6)	
H6	0.8453	0.4644	0.3769	0.060*	
C7	0.8148 (2)	0.2577 (3)	0.5473 (2)	0.0541 (6)	
H7A	0.7510	0.3341	0.5495	0.065*	
H7B	0.8506	0.2456	0.6335	0.065*	
C8	0.6212 (2)	0.1615 (2)	0.3776 (2)	0.0442 (5)	
C9	0.4869 (2)	0.1511 (2)	0.2191 (2)	0.0462 (6)	
N1	0.5965 (2)	0.29303 (18)	0.3524 (2)	0.0541 (5)	
N2	0.5088 (2)	0.28358 (17)	0.2498 (2)	0.0539 (5)	
H21	0.473 (3)	0.3613 (18)	0.216 (2)	0.081*	
N3	0.55663 (18)	0.06938 (16)	0.29881 (19)	0.0460 (5)	
N4	0.4055 (2)	0.1079 (2)	0.1236 (2)	0.0665 (6)	
H41	0.404 (3)	0.0173 (12)	0.107 (3)	0.100*	
H42	0.368 (3)	0.167 (3)	0.068 (2)	0.100*	
S1	0.72492 (7)	0.10082 (6)	0.50093 (7)	0.0582 (2)	

### Atomic displacement parameters (Å^2^)

**Table:**

	*U*^11^	*U*^22^	*U*^33^	*U*^12^	*U*^13^	*U*^23^
C1	0.0478 (13)	0.0408 (11)	0.0385 (11)	−0.0047 (9)	−0.0002 (10)	−0.0064 (10)
C2	0.0548 (15)	0.0433 (13)	0.0548 (14)	0.0000 (10)	−0.0025 (12)	0.0058 (11)
C3	0.0481 (15)	0.0580 (15)	0.0735 (16)	0.0027 (12)	0.0074 (13)	0.0032 (13)
C4	0.0516 (16)	0.0676 (17)	0.0625 (15)	−0.0123 (13)	0.0078 (12)	0.0057 (14)
C5	0.0657 (18)	0.0511 (14)	0.0522 (14)	−0.0083 (12)	−0.0014 (13)	0.0119 (12)
C6	0.0508 (14)	0.0453 (12)	0.0543 (13)	0.0016 (10)	−0.0055 (11)	0.0007 (11)
C7	0.0587 (16)	0.0511 (13)	0.0525 (13)	−0.0024 (11)	0.0059 (12)	−0.0027 (11)
C8	0.0382 (12)	0.0273 (10)	0.0672 (14)	0.0003 (9)	0.0137 (11)	0.0022 (10)
C9	0.0393 (12)	0.0266 (10)	0.0728 (15)	−0.0016 (9)	0.0111 (11)	0.0016 (11)
N1	0.0525 (13)	0.0264 (9)	0.0834 (14)	0.0016 (8)	−0.0020 (11)	−0.0027 (9)
N2	0.0495 (12)	0.0245 (9)	0.0876 (15)	0.0002 (8)	−0.0015 (11)	0.0035 (9)
N3	0.0400 (10)	0.0226 (8)	0.0753 (13)	−0.0012 (7)	0.0092 (9)	0.0002 (9)
N4	0.0709 (16)	0.0374 (11)	0.0912 (17)	−0.0030 (10)	−0.0166 (13)	0.0004 (12)
S1	0.0576 (4)	0.0398 (3)	0.0774 (5)	−0.0023 (3)	0.0053 (3)	0.0130 (3)

### Geometric parameters (Å, °)

**Table:**

C1—C2	1.384 (3)	C7—H7A	0.9700
C1—C6	1.386 (3)	C7—H7B	0.9700
C1—C7	1.509 (3)	C8—N1	1.317 (3)
C2—C3	1.377 (3)	C8—N3	1.365 (3)
C2—H2	0.9300	C8—S1	1.740 (2)
C3—C4	1.376 (3)	C9—N3	1.333 (3)
C3—H3	0.9300	C9—N2	1.334 (3)
C4—C5	1.371 (4)	C9—N4	1.342 (3)
C4—H4	0.9300	N1—N2	1.375 (3)
C5—C6	1.380 (3)	N2—H21	0.900 (10)
C5—H5	0.9300	N4—H42	0.892 (10)
C6—H6	0.9300	N4—H41	0.889 (10)
C7—S1	1.817 (2)	N4—H42	0.892 (10)
			
C2—C1—C6	118.5 (2)	C1—C7—H7B	108.8
C2—C1—C7	119.8 (2)	S1—C7—H7B	108.8
C6—C1—C7	121.7 (2)	H7A—C7—H7B	107.6
C3—C2—C1	121.0 (2)	N1—C8—N3	114.9 (2)
C3—C2—H2	119.5	N1—C8—S1	125.35 (18)
C1—C2—H2	119.5	N3—C8—S1	119.75 (15)
C4—C3—C2	119.8 (2)	N3—C9—N2	109.5 (2)
C4—C3—H3	120.1	N3—C9—N4	125.7 (2)
C2—C3—H3	120.1	N2—C9—N4	124.8 (2)
C5—C4—C3	120.0 (2)	C8—N1—N2	101.91 (18)
C5—C4—H4	120.0	C9—N2—N1	110.48 (19)
C3—C4—H4	120.0	C9—N2—H21	129.8 (18)
C4—C5—C6	120.1 (2)	N1—N2—H21	119.7 (17)
C4—C5—H5	119.9	C9—N3—C8	103.18 (17)
C6—C5—H5	119.9	H42—N4—C9	122 (2)
C5—C6—C1	120.6 (2)	H42—N4—H41	120 (3)
C5—C6—H6	119.7	C9—N4—H41	117 (2)
C1—C6—H6	119.7	H42—N4—H42	0(2)
C1—C7—S1	113.99 (16)	C9—N4—H42	122 (2)
C1—C7—H7A	108.8	H41—N4—H42	120 (3)
S1—C7—H7A	108.8	C8—S1—C7	101.62 (11)

### Hydrogen-bond geometry (Å, °)

**Table:**

*D*—H···*A*	*D*—H	H···*A*	*D*···*A*	*D*—H···*A*
N4—H41···N1^i^	0.89 (1)	2.20 (2)	3.044 (3)	158 (3)
N2—H21···N3^ii^	0.90 (1)	2.03 (2)	2.873 (2)	155 (2)

Symmetry codes: (i) −*x*+1, *y*−1/2, −*z*+1/2; (ii) −*x*+1, *y*+1/2, −*z*+1/2.

## References

[bb1] El-Sagheer, A. H. & Brown, T. (2011). *Chem. Commun.* **47**, 12057–12058. 10.1039/c1cc14316f 21961113

[bb2] Higashi, T. (1995). *ABSCOR* Rigaku Corporation, Tokyo, Japan.

[bb3] Paulvannan, K., Hale, R., Sedehi, D. & Chen, T. (2001). *Tetrahedron*, **57**, 9677–9682.

[bb4] Rigaku (1998). *RAPID-AUTO* Rigaku Corporation, Tokyo, Japan.

[bb5] Rigaku/MSC (2002). *CrystalStructure* Rigaku/MSC Inc., The Woodlands, Texas, USA.

[bb6] Sheldrick, G. M. (2008). *Acta Cryst.* A**64**, 112–122. 10.1107/S0108767307043930 18156677

